# Annotations

**Published:** 1904-03-26

**Authors:** 


### Annotations.

### Arsenic in Cream of Tartar.

There is no need to be an alarmist in order to admit that the discovery of arsenic, even in small quantities, in various articles previously unsuspected of the presence of this powerful mineral, is a somewhat disquieting fact. The latest substance to obtain this bad eminence is cream of tartar, which, in addition to its medicinal use, is largely employed in baking and for other domestic purposes. From prosecutions which have been lately conducted in various parts of the country, it appears that arsenic is being detected in cream of tartar in amounts ranging from 1-30th to 1-10th of a grain per pound. The source of the contamination is for the present unknown. Cream of tartar comes to this country from the various vine-growing districts of Southern Europe, and it is suggested that the grapes may receive a small amount of arsenic from the practice of washing the vines, as the growers are in the habit of doing, with some arsenical preparation. However this may be, it is satisfactory to find that the Sale of Food and Drugs Act is carried out in such a manner as to recognise even a quite unexpected form of adulteration. There have been instances in which the zeal of the public analyst has been more conspicuous than his discretion, but on the whole the Act has conferred vast benefits upon the public. The present case is an example of this benefit. Naturally the vendor who has sold the impure article tries to minimise the effect of the impurity, and it may be true, as was said in one of the recent cases, that "a person would have to eat 288 scones" in order to take 1-20th of a grain of arsenic. But, though it is sometimes hard on the individual vendor who is entirely guiltless of any intentional offence, we hope the law will be steadily put in force. It is only by prosecuting the seller of arsenicated cream of tartar that an earnest demand will be created for a supply of the pure article. Practical bakers and commercial travellers may offer what "opinion" they will regarding the harmlessness of small doses of arsenic, but the British public wisely prefers its cream of tartar and its "scones" uncontaminated with mineral poison.

### Medicine and Law.

It is an undeniable fact that the dignity of the law occasionally has to maintain itself against some odds when questions involving pathological or physiological considerations arise in court. More than one forensic definition, dating from a remote and unenlightened past, is even now accepted, although it may be quite at variance with modern scientific experience. Notably is this so in matters where the mental condition of an individual is under examination. The legal test which is applied to the possibly insane criminal, namely, his power or otherwise to distinguish between right and wrong, is not in accord with the present-day knowledge of mental derangements. The alienist would make the distinction rest rather upon the ground of the power to choose the correct course than the ability to perceive it. The difference between the two points of view is as wide as it is important. That there should be such discrepancies between forensic definitions and modern medical science is but natural, seeing that the latter has been revolutionised in the course of the last fifty years; and it is by no means to be supposed that the former would be justified, even were such a thing possible, in adapting itself to every modern change in medical opinion the moment it occurs. Indeed, there is every reason why the attitude of the legal profession towards medical doctrines should be one of suspicion, because the doctors' creed is for ever changing and the tenets of one individual member of the profession are often combated by his neighbour. But making every allowance for the practical difficulties in the way of a satisfactory solution, we are still of opinion that the time has come when modern advances of medical knowledge should meet with a proper recognition in the legal code.

### The Disposal of London Sewage.

Owing to allegations which had been made to the effect that shellfish layings in the estuary of the Thames were exposed to contamination by the sewage effluents from the Metropolis, the London County Council have recently made an inquiry into the matter, and their conclusions now appear in the form of a report. Some notion of the magnitude of the problem of disposal of London sewage may be gathered from the statement that the amount treated during 1903 was 94,808,085,000 gallons. The whole of this is discharged, after sedimentation with lime and sulphate of iron, at the two outfalls at Barking and Erith. The sediment, or sludge, is carried out to sea and deposited in Barrow Deep—a channel which is practically the open sea, but which is not used for navigation. In order to investigate the condition of the river below the outfalls, samples were taken at low water at various points. The specimens were tested bacteriologically and for the degree of aeration. The latter showed that the degree of aeration increased steadily with the increase of distance below the outfalls. The number of bacteria per cubic centimetre of water which averaged 5,125 immediately below the outfalls progressively diminished until 3½ miles below the Nore they stood at the small figure of 171. So far as gas-forming bacteria were concerned they were only found in seven out of the 45 samples taken, and none were found at a distance of more than 27 miles below the sewage outfalls. That such a satisfactory result should be achieved in dealing with the gigantic problem redounds very much to the credit of the London County Council. Nevertheless, after reading the report referred to, we do not feel altogether reassured with regard to shellfish in the estuary of the river Thames; the very fact that gas-producing bacteria were found at a distance of 27 miles from the sewage outfalls should be a warning of danger.

